# Dinucleotide controlled null models for comparative RNA gene prediction

**DOI:** 10.1186/1471-2105-9-248

**Published:** 2008-05-27

**Authors:** Tanja Gesell, Stefan Washietl

**Affiliations:** 1 Center for Integrative Bioinformatics Vienna, Max F. Perutz Laboratories, Dr. Bohr-Gasse 9, A-1030 Vienna, Austria; 2 University of Vienna, Austria; 3 Medical University of Vienna, Austria; 4 University of Veterinary Medicine, Vienna, Austria; 5 Institute for Theoretical Chemistry, University of Vienna, Währingerstraße 17, 1090 Vienna, Austria; 6 EMBL-European Bioinformatics Institute, Wellcome Trust Genome Campus, Hinxton, Cambridge CB10 1SD, UK

## Abstract

**Background:**

Comparative prediction of RNA structures can be used to identify functional noncoding RNAs in genomic screens. It was shown recently by Babak *et al*. [BMC Bioinformatics. 8:33] that RNA gene prediction programs can be biased by the genomic dinucleotide content, in particular those programs using a thermodynamic folding model including stacking energies. As a consequence, there is need for dinucleotide-preserving control strategies to assess the significance of such predictions. While there have been randomization algorithms for single sequences for many years, the problem has remained challenging for multiple alignments and there is currently no algorithm available.

**Results:**

We present a program called SISSIz that simulates multiple alignments of a given average dinucleotide content. Meeting additional requirements of an accurate null model, the randomized alignments are on average of the same sequence diversity and preserve local conservation and gap patterns. We make use of a phylogenetic substitution model that includes overlapping dependencies and site-specific rates. Using fast heuristics and a distance based approach, a tree is estimated under this model which is used to guide the simulations. The new algorithm is tested on vertebrate genomic alignments and the effect on RNA structure predictions is studied. In addition, we directly combined the new null model with the RNAalifold consensus folding algorithm giving a new variant of a thermodynamic structure based RNA gene finding program that is not biased by the dinucleotide content.

**Conclusion:**

SISSIz implements an efficient algorithm to randomize multiple alignments preserving dinucleotide content. It can be used to get more accurate estimates of false positive rates of existing programs, to produce negative controls for the training of machine learning based programs, or as standalone RNA gene finding program. Other applications in comparative genomics that require randomization of multiple alignments can be considered.

**Availability:**

SISSIz is available as open source C code that can be compiled for every major platform and downloaded here: .

## Background

Comparative genome analysis is currently the most widely used strategy to detect and annotate noncoding RNAs (ncRNAs) [1,2]. In the past few years a series of different algorithms have been developed that predict functional ncRNAs on the basis of conserved secondary structure [3-10]. Some of these methods have been used to predict novel ncRNAs on a genome wide scale [7,11-14]. In combination with experimental verification (microarray, RT-PCR, Northern blot) these methods could successfully uncover many examples of novel ncRNAs [15-20]. However, in particular in large vertebrate genomes the signal-to-noise ratio of true predictions and false positives is thought to be relatively low [20]. In a recent paper, Babak and colleagues demonstrated that comparative ncRNA gene finders are strongly biased by the genomic dinucleotide content leading to an excess of false predictions [21]. Especially methods that are based on a thermodynamic folding model are sensitive to this effect: In the so-called nearest neighbour model, energies are not assigned to single base-pairs but rather to neighbouring base-pairs that stack on each other. As a consequence, the folding stability of genomic sequences does not only depend on the monunucleotide content but also the dinucleotide content.

To assess the significance of predicted structures, e.g. to estimate the false discovery rate in a genomic screen for ncRNAs, one should therefore compare the genomic predictions to the results obtained on randomized data with the same dinucleotide content. In the case of single sequences, there are well known and widely used algorithms to generate dinucleotide controlled random sequences either by shuffling or first order Markov chain simulation [22,23]. However, there is currently no algorithm to randomize multiple sequence alignments preserving the dinucleotide content. Babak and colleagues [21] added the conservation of dinucleotides as an additional constraint to the commonly used (mononucleotide) shuffling algorithm shuffle-aln.pl [5] and applied it to pairwise alignments. Their approach corresponds to a heuristic used in reference 24, that is very inefficient as only a small subspace of the whole permutation space is covered. The heuristic exchanges only positions that have the same neighbours left and right. For the short sequence ACAGCCAA for example not a single permutation can be found that way. However, there are 11 such permutations according to the Altschul & Erikson algorithm [22]. But even a more efficient shuffling algorithm will soon run into difficulties on multiple alignments. Unless two neighbouring columns are 100% conserved, there are several different dinucleotide pairs in these columns. It is therefore impossible to exactly preserve the dinucleotide content as in the single sequence case.

In this paper, we address the problem in a different way. In analogy to a first order Markov model for single sequences, we simulate alignments of a given dinucleotide content. We present a substitution model that captures the neighbour dependencies and all other relevant alignment features. We describe a time efficient way to estimate a tree under this model that we use as a guide to simulate alignments of the desired properties. This new control strategy is tested on genomic alignments and the effect on thermodynamic RNA structure predictions is studied.

## Results

### Requirements for an accurate null model

An optimal null model preserves all the features of the original data with the exception of the signal under question that needs to be removed efficiently. In our case, the data are multiple alignments of homologous sequences and the signal of interest is an evolved RNA secondary structure. Correlations arising from base-pairing patterns need to be removed. Currently, alignments are usually randomized by shuffling the alignment columns (see ref. 5 for a discussion of this method). Although the shuffling approach has its limitations and considering dinucleotides seems difficult, it is an appealing approach because it is relatively simple, fast, and extremely conservative. Changing the order of the columns does not change the mutational patterns within the columns and thus the underlying phylogenetic tree is exactly preserved.

In this paper we attempt to simulate new alignments from scratch. Even the most sophisticated model cannot capture all evolutionary processes and therefore a simulation approach will inevitably change the original data more than shuffling does. So much care has to be taken to preserve all the relevant characteristics of the data. To qualitatively assess the most important parameters that need to be considered in our model, we performed a series of simulation experiments. Using a simple tree with four taxa we simulated alignments under the HKY evolutionary model [25]. We systematically varied model and tree parameters to study how they affect thermodynamic RNA consensus structure predictions in the alignment. We used RNAalifold [26] to predict consensus secondary structures which is the basis of the AlifoldZ [5] and RNAz [6] gene finders.

**Figure 1:**
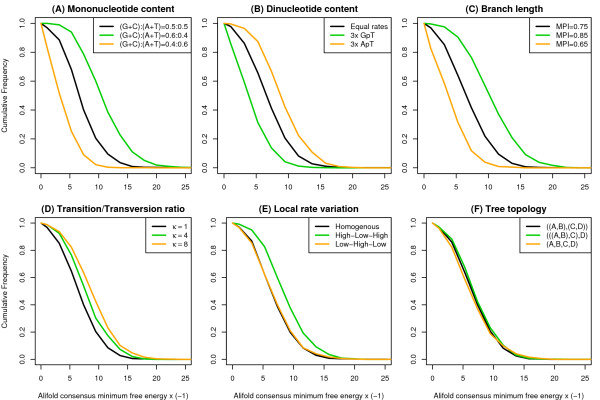
**Parameters effecting thermodynamic consensus RNA structure predictions.** As a basic parameter set we used equal base frequencies of 0.25, a transition/transversion rate ratio *κ *= 1, and the following tree ((A:0.09,B:0.09):0.09,(C:0.09,D:0.09):0.09) One parameter was varied at a time while others were kept constant. If necessary branch lengths were adjusted to keep a mean pairwise sequence identity (MPI) of 0.75 ± 0.01. 1000 alignments of length 80 were simulated under each condition. Cumulative histograms for the RNAalifold consensus folding energies are shown. Please note that we plot negative minimum free energies, i.e. higher values correspond to more stable folds. (**A**) Base frequencies were varied to get high and low G+C content. (**B**) Two specific dinucleotide frequencies were elevated 3-fold while the mononucleotide content was kept constant. (**C**) Branch lengths were equally scaled to produce alignments with lower or higher MPI identity than for the basic tree. (**D**) The transition/transversion rate ratio was varied. *κ *= 1 means equal rates, while *κ *> 1 gives more transition than transversions. (**E**) The alignment of size 80 was divided into a central block of 40 and two anking regions of 20. We set 100% conservation in the central block and low conservation in the anks (rate "high-low-high") and the other way round ("low-high-low"). The total average MPI was always 0.75. (**F**) We tested all possible topologies of this 4 taxa tree and adjusted the branch lengths to give a MPI of 0.75. For one given topology, all the branch lengths were of the same length.

Not surprisingly, base composition is one of the parameters affecting the predicted folding energies strongest (Fig. 1A). High G+C content leads to more stable RNA predictions, while high A+T content gives less stable predictions. As mentioned in the introduction and in fact the main motivation of this paper, also dinucleotide content affects folding energies. We used our simulation algorithm that is described below to simulate alignments of the same mononucleotide content but varying dinucleotide content. Fig. 1B shows for example that a three times enriched ApT content lead to more stable predictions. The excess of some other dinucleotides like for example GpT can cause the opposite effect leading to less stable predictions.

Another major parameter that needs to be controlled is the sequence diversity of the alignment. Variation of the branch lengths of the tree gives alignments with different sequence diversity which we usually measure as the mean pairwise sequence identity (MPI, also sometimes refered to as average pairwise sequence identity APSI). High diversity (i.e. low MPI) makes it difficult to predict a consensus structure if there is no selection pressure for it. On the other hand, almost perfectly conserved sequences fold readily in some random structure even if there is no natural RNA structure present. Therefore we observe a strong dependency on the MPI (Fig. 1C).

One well known characteristic of natural mutation processes are the different rates for transitions and transversions [27]. Interestingly, this also affects the consensus structure predictions. A model with equal transition/transversion rates (parameter *κ *= 1 in the HKY model) gives less stable predictions than a model with more realistic rates (e.g *κ *= 4, Fig. 1D). This parameter affects the type of column patterns observed in the simulated alignments which in turn affects how well they can form consensus base pairs.

Natural mutation processes are not homogeneous across all sites, in particular in functional genomic regions. It was observed previously that mutation patterns within an alignment can affect structure predictions [5]. For example, an alignment containing a 100% conserved block with low mutation rate that is flanked by highly divergent regions of high mutation rate can have different folding energies compared to an alignment with homogeneous rates but the same overall MPI (Fig. 1E). The same is true for patterns of insertions and deletions which was also already discussed in reference 5 and which we do not show here explicitly again.

We also tested the effect of different tree topologies, but did not find a significant influence of this parameter at least in our four taxa example.

Taken together, an accurate randomization procedure needs to generate alignments that preserve (i) mono- and dinucleotide content, (ii) mean pairwise sequence identity, (iii) transition/transversion rate ratio (iv) site-specific mutation rates, and (v) gap patterns.

In the next section we describe a model that is capable of simulating alignments under these constraints.

### Algorithm

#### Model

Sequence evolution is usually described by a time-continuous Markov process [27,28]. The most commonly used models assume that all sites of a sequence evolve independently from each other rendering it impossible to model dinucleotide dependencies between neighbouring pairs. Various evolutionary models have been proposed in the past years to overcome this limitation [29-36]. We make use of the recently introduced framework called SISSI (SImulating Site-Specific Interactions). SISSI allows to define site dependencies of arbitrary complexity in the form of a "neighbourhood system" that also may include overlapping dependencies [37]. Given the requirements of our specific problem, we extended and simplified several aspects of SISSI as necessary.

**Figure 2:**
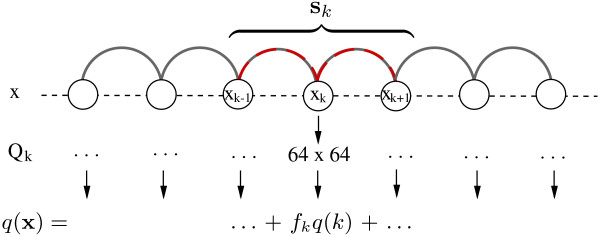
**Site dependencies for overlapping dinucleotides (red-gray):** The substitution process of a given nucleotide *x*_*k *_at site *k *by another one depends on the states *x*_*k*-1_, *x*_*k*_, *x*_*k*+1_, the subsequence **s**_*k*_. *Q*_*k *_has the dimension 64 × 64, where only one mutation is allowed at the current site *k*. The substitution rate for the whole sequence *q*(**x**) is the sum of each rate *q*(*k*) = *Q*_*k*_(**s**_*k*_, **s**_*k*_) multiplied with a site-specific scaling factor *f*_*k*_, with *k *= 1, ..., *l*.

Following the general framework of SISSI, we introduce a site-specific rate matrix *Q*_*k *_for every site *k *= 1, ..., *l *in a sequence **x **= (x _1_, ..., x _l_). This matrix defines the substitution process at site *k*, where the substitution of a given nucleotide *x*_*k *_∈ A = {A,C,G,U} by another one depends on the states *x*_*k*-1_, *x*_*k*_, *x*_*k*+1 _(Fig. 2).

Thus, the instantaneous rate matrix *Q*_*k *_has the dimension |A|^3 ^× |A|^3 ^= 64 × 64. The stationary distribution of *Q*_*k *_determines the equilibrium dinucleotide content of our system (see the next section for how the required trinucleotide frequencies of *Q*_*k *_are calculated from the dinucleotide frequencies).

To be able to control the transition/transversion rate ratio and the site-specific mutation rates, we have to add two additional parameters. Let **s**_*k *_= (*x*_*k*-1_, *x*_*k*_, *x*_*k*+1_) represent the current triplet of sequence **x **and **y **= (*y*_1_, *y*_2_, *y*_3_) another triplet in A^3^. First, we introduce a general parameter *r*(**s**_*k*_, **y**) ≥ 0 to incorporate the additional mechanistic rates. Second, we introduce a site-specific scaling factor *f*_*k *_with *k *= 1, ..., *l*, such that:

(1) $$\frac{1}{l}\cdot \sum_{1}^{l}f_{k}=1.$$

We impose the usual restriction, that only one substitution per unit time is admissible [38,39]. Moreover, *Q*_*k *_only allows for substitutions at site *k*. The diagonal elements of our instantaneous rate matrix *Q*_*k *_are defined by the mathematical requirement that the sum of each row is zero.

The entries of *Q*_*k *_are thus given by

(2) $$Q_{k}(s_{k},y)=f_{k}\cdot \{\begin{array}{ll} r_{\left(s_{k},y\right)}\cdot \pi_{k}(y) & \text{if }H(s_{k},y)=1\text{ and }x_{k}\neq y_{2}\\ -\sum_{\begin{array}{l} z\in A^{3}\\ z\neq s_{k} \end{array}}Q_{k}(s_{k},z) & \text{if }H(s_{k},y)=0\\ 0 & \text{otherwise} \end{array}$$

where *π*_*k*_(**y**) is the stationary frequency of **y **and the Hamming distance *H*(**s**_*k*_, **y**) counts the number of differences between the sites of the triplets **s**_*k*_and **y**.

In principle, we can choose any rate for the parameter *r*(**s**_*k*_, **y**). However, based on the requirement that we want to use the counted dinucleotide content as the stationary distribution, we choose *r*(**s**_*k*_, **y**) so that the model becomes reversible. Any parameter of the commonly used independent nucleotide substitution models, like HKY [25] or the general time-reversible model GTR [40] can be chosen for *r*(**s**_*k*_, **y**). For our application, we use a transition/transversion rate ratio and set *r*(**s**_*k*_, **y**)= *κ *for transitions and *r*(**s**_*k*_, **y**) = 1 for transversions.

The restriction that a substitution is only possible at site *k *leads to sparse rate matrices. *Q*_*k *_has only |A|^4 ^non-zero entries. Hence, we can write *Q*_*k *_in the form of 16 submatrices, which describe the admissible substitutions for site *k *depending on the left *y*_1 _and right *y*_3 _neighbours,

(3) $$\begin{array}{cc} \begin{array}{c} y_{1}\text{A}y_{3}\\ y_{1}\text{C}y_{3}\\ y_{1}\text{G}y_{3}\\ y_{1}\text{U}y_{3} \end{array} & \begin{array}{c} \begin{array}{cccc} y_{1}\text{A}y_{3} & y_{1}\text{C}y_{3} & y_{1}\text{G}y_{3} & y_{1}\text{U}y_{3} \end{array}\\ \left(\begin{array}{cccc} {}* & \pi_{y_{1}\text{C}y_{3}} & \kappa \pi_{y_{1}\text{G}y_{3}} & \pi_{y_{1}\text{U}y_{3}}\\ \pi_{y_{1}\text{A}y_{3}} & * & \pi_{y_{1}\text{G}y_{3}} & \kappa \pi_{y_{1}\text{U}y_{3}}\\ \kappa \pi_{y_{1}\text{A}y_{3}} & \pi_{y_{1}\text{C}y_{3}} & * & \pi_{y_{1}\text{U}y_{3}}\\ \pi_{y_{1}\text{A}y_{3}} & \kappa \pi_{y_{1}\text{C}y_{3}} & \pi_{y_{1}\text{G}y_{3}} & * \end{array}\right) \end{array} \end{array}$$

Finally, we scale *Q*_*k *_such that the number of substitutions *d*_*k *_equals 1:

(4) $$d_{k}=-\sum_{z\in A^{3}}\pi_{k}(z)\cdot Q_{k}(z,z)=1.$$

and thus the total instantaneous substitution rate for a sequence **x **can be written as the sum over each rate *Q*_*k*_(**s**_*k*_, **s**_*k*_) multiplied with the site-specific scaling factor *f*_*k*_, with *k *= 1, ..., *l *(Fig. 2),

(5) $$q(x)=-\sum_{k=1}^{l}f_{k}\cdot Q_{k}(s_{k},s_{k}).$$

Without dependencies on the neighbours, *Q*_*k *_is of dimension 4 × 4 and the model reduces essentially to a HKY model with a specific rate for each site. We use this mononucleotide variant later in this paper for testing and comparison to the dinucleotide model.

#### Simulation

For the simulation process, we essentially used the same algorithm described previously [37] with some modifications. During the simulation process, we pick a site *k *with the relative mutability

(6) $$P(k)=\frac{\left|f_{k}\cdot Q_{k}(s_{k},s_{k})\right|}{q(x)},$$

and for the chosen site *k*, the nucleotide *x*_*k *_will replaced by a new nucleotide *y*_2 _∈ A from the corresponding triplet **y **with probability:

(7) $$P(x_{k}\to y_{2})=\frac{f_{k}\cdot Q_{k}(s_{k},y)}{\left|f_{k}\cdot Q_{k}(s_{k},s_{k})\right|}=\frac{Q_{k}(s_{k},y)}{\left|Q_{k}(s_{k},s_{k})\right|}$$

In the most general SISSI framework *Q*_*k *_needs to be updated for all *k *sites every time one nucleotide in **x **is substituted. However, in our special case we can use the same instantaneous rate matrix *Q*_*k *_for each site with special conditions for *r*(**s**_*k*_, **y**). As a consequence, we can fix *q*(**x**) and do not need to sum over each rate of the site, which improves the running time of the algorithm.

#### Parameter estimation

The idea of our randomization procedure is to estimate a tree under the model described in the previous section and simulate sequences along this tree. Ideally, all parameters are estimated simultaneously within a maximum likelihood framework. One problem is the high number of parameters since we want to estimate a specific rate for each site. A more fundamental issue is, however, that our model includes overlapping dependencies which breaks the independence assumption necessary for basic maximum likelihood estimation. Other possible techniques like Markov chain Monte Carlo in a Bayesian framework are not a viable alternative either. Speed is a critical issue as the algorithm is meant to be applied to data on a genome wide scale.

Facing these difficulties, we have developed heuristic approximations to estimate the parameters and use a distance based approach to estimate the tree. The method is fast and yet surprisingly accurate for our application.

#### Equilibrium frequencies

The stationary frequencies of our model are set in a way that in equilibrium we obtain a dinucleotide frequency that is the same as the dinucleotide content of the alignment to be randomized. To this end, we first count the dinucleotide frequencies as an average of all sequences in the original alignment (see Methods on how gaps are treated). Then, we calculate the corresponding trinucleotide frequencies needed for *Q*_*k *_as a function of the single and dinucleotide frequencies using an approximation based on simple conditional probabilities [30,32]:

(8) $$\pi (\alpha \beta \gamma )=\frac{\pi (\alpha \beta )\pi (\beta \gamma )}{\pi (\beta )}$$

where *π*(*αβγ*) are the trinucleotide frequencies, *π*(*αβ*) and *π*(*βγ*) the counted dinucleotide frequencies and *π*(*β*) = Σ_*α *_*π*(*αβ*) = Σ_*α *_*π*(*βα*) with *α*, *β*, *γ *∈ {A, C, G, U}.

**Figure 3:**
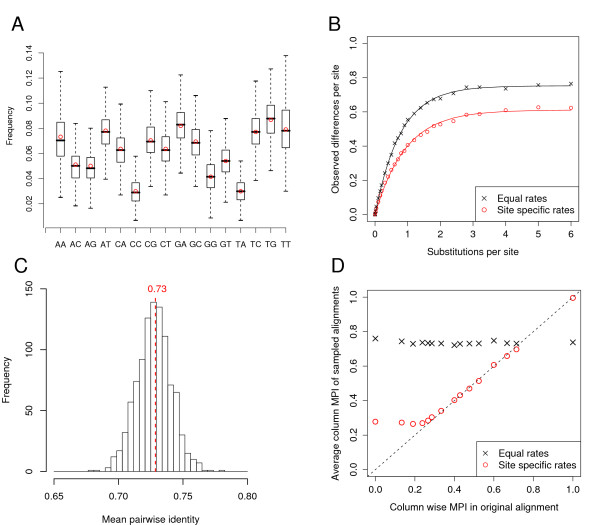
**Key concepts of the algorithm shown on an example alignment of 5.8S rRNA.** (**A**) Distribution of dinucleotide frequencies of 1000 simulated alignments are shown as box-plots (the line in the box indicates the median, the borders of the box the 25th and 75th quartile, and the dotted lines 1.5× the interquartile range). Red circles show the frequencies observed in the original alignment. (**B**) Relationship between the number of substitutions and observed differences empirically determined by sampling of 25 points. Each point shows the average of 10 simulations. Note that the short distances are sampled more densely. These settings are the default values in our program and used throughout the paper. (**C**) Distribution of mean pairwise identities for 1000 random samples. The MPI of the original alignment is shown in red. (**D**) Comparison of site-wise MPIs in the original alignment and the average of the corresponding sites of 1000 random alignments.

Fig. 3A shows an example of the dinucleotide frequency distribution of 1000 simulated alignments. We counted the dinucleotide frequencies of an alignment of 7 5.8 rRNA sequences and set the trinucleotide parameters of our model accordingly. On average, we get the same dinucleotide frequencies in the simulated alignments as in the original one.

#### Distances and tree construction

To build a distance based tree, we first have to estimate the number of substitutions that have taken place between two sequences. In other words, we have to estimate the genetic or evolutionary distance *d *from the Hamming distances *p *under our model. Both distances are different because back mutations have taken place that are not directly visible. To estimate the relationship between *d *and *p*, we simulate sequence pairs separated by different branch lengths *d *and calculate the corresponding Hamming distances *p *(Fig. 3B). We fit an exponential function to this curve:

(9) $$p=\hat{a}\cdot (1-e^{\hat{b}\cdot d})$$

Using this function, all pairwise distances are calculated for the sequences in the original alignment. From this distance matrix a tree is constructed using the BIONJ algorithm [41]. BIONJ is a variant of the well known neighbour joining algorithm and currently one of the most accurate algorithms for distance based tree building.

Given that the distances and the tree are accurately estimated, we observe on average the same mean pairwise identity in the simulated alignment as in the original one. Fig. 3C shows the distribution of MPIs of 1000 simulations of our example rRNA alignment. The average MPI of the simulations is exactly the same as the MPI 0.73 of the original alignment.

#### Site-specific rates

Setting different mutation rates at different sites gives us the possibility to preserve natural mutation patterns of the original alignment. The problem of finding accurate site-specific rates is illustrated in Fig. 3D. For each site in the alignment, the MPI of this site is plotted against the average MPI observed in the simulated alignments on the same site. If we consider equal rates for all sites, each site will have the same average MPI which is of course equal to the overall MPI of 0.73 of the whole alignment. Ideally, the average MPI for each simulated site is the same as the original MPI at this site. In this case, the points in the plot are on a diagonal indicating that we have found accurate estimates for the rates.

Simple estimates for site-specific rates in combination with distance based trees have been described previously [42]. The method includes fits to a gamma distribution which requires data of at least 1000 nucleotides and 30 sequences to get reasonable results. Here we use a different approach that also gives good results for smaller alignments.

The substitution rate at a site is of course related to the observed sequence diversity at this site. If a site is highly conserved the rate is low, whereas high sequence diversity indicates a high mutation rate. So in a first step, we calculate the average number of pairwise differences ⟨*p*_*k*_⟩ for each site *k *in the alignment with *n *sequences:

(10) $$\begin{array}{cc} \langle p_{k}\rangle =\frac{2}{n(n-1)}\sum_{i}^{n}\sum_{j>i}^{n}\delta_{ij}^{k}; & \text{with }\delta_{ij}^{k}=\{\begin{array}{ll} 1 & \text{if nucleotides in sequences }i,j\text{ differ at site }k\\ 0 & \text{otherwise} \end{array} \end{array}$$

⟨*p*_*k*_⟩ are observed differences ignoring multiple substitutions. If we naively choose our rates proportional to ⟨*p*_*k*_⟩ we would underestimate high rates while overestimating low rates. We therefore use the relationship in equation 9 to correct for this effect and calculate estimates ${\hat{f}}_{k}$ for the rates at site *k *as follows:

(11) $${\hat{f}}_{k}=\frac{1}{\hat{b}}\cdot \ln\left(1-\frac{\langle p_{k}\rangle }{\hat{a}}\right)$$

It must be pointed out that the site-specific rates change the relationship between genetic distance and observed differences (Fig. 3B). For correcting the site-specific rates we use the estimates for $\hat{a}$ and $\hat{b}$ from our model *without *site-specific rates. So this is only an approximation and one could think about iteratively refining the estimates. However, we found that this approach already yields accurate rates within one step as can be seen in Fig. 3D. Using the model with site-specific rates, the simulated alignments have on average almost exactly the same site-wise MPI as the original one.

The reader will notice that the first three points deviate from the diagonal. This illustrates a limitation of our method. With our simulation procedure we can on average only reach the level of saturation even if we use very high rates. It is possible, however, that the original data contains sites below the level of saturation. For example in a four way alignment a column can be ACGT, i.e. MPI = 0. However, we cannot simulate on average columns with MPI = 0, since the MPI is bounded below by zero and our simulations will always contain columns with MPI > 0. In practice this does not seem to cause any obvious problems in particular when we have many sequences where it is unlikely to see columns below saturation.

#### Gaps

Gaps have been ignored completely so far. There are evolutionary models including deletions and insertions [43-47] and, in principle, it would be possible to combine the insertion-deletion dynamics with our model. However, this does not appear practical in our case. Existing algorithms for joint estimation of phylogenies and alignments are not only very time-consuming [47], it also seems difficult to estimate reasonable indel model parameters on relatively short alignment blocks which hold only little information. Moreover, alignment programs produce gap patterns that do not necessarily reflect phylogenetically reasonable insertion/deletion events and thus cannot always be captured by an idealized model that is motivated by evolutionary processes and ignores algorithmic idiosyncrasies of alignment programs.

So we follow here a very pragmatic strategy that has also been used previously [5]: We keep exactly the same gap pattern in our randomized alignments as in the original alignment. To this end, we simply treat gaps as missing data and simulate nucleotide characters for the gapped positions. This is done in a way that the overall characteristics are not changed when they are replaced with gaps again at the end (see Methods for details).

#### Transition/transversion rate ratio

The transition/transversion rate ratio *κ *is a parameter in our model that cannot be simply counted as in the case of the dinucleotide frequencies, or empirically determined like the branch lengths. Given that the influence of this parameter is not that critical as for example the branch length or base composition (see Fig. 1), one possibility might be to use a fixed transition/transversion ratio if a reasonable average value is known for the genome at hand. Alternatively, we found that a good estimate can be obtained by using maximum likelihood on an independent mononucleotide model. We used here the HKY model with -distributed rates which is closest to our dinucleotide model.

#### Putting it together

**Figure 4:**
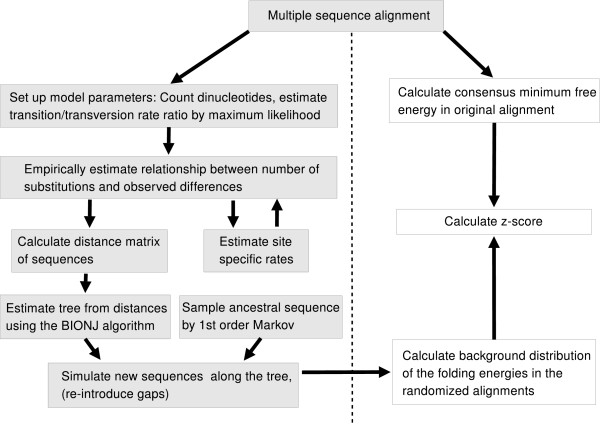
**Overview of the algorithm.** Left: The steps of the randomization procedure are shown. Right: In combination with RNAalifold consensus folding the randomization procedure can be used to calculate *z*-scores and to predict significant RNA structures. See text for details.

**Figure 5:**
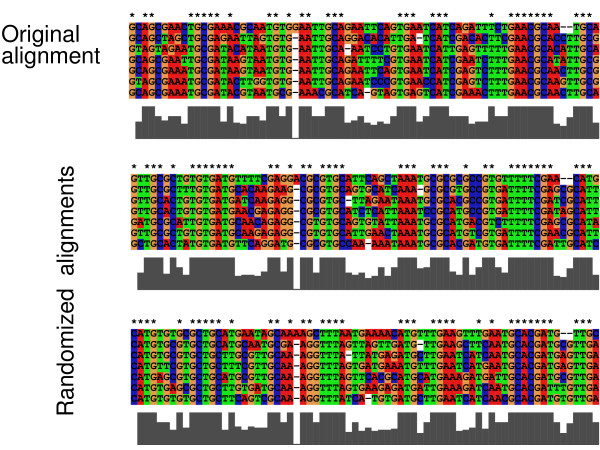
**Example of randomized alignments.** Part of the example alignment used in Fig. 3 are shown. The grey bars indicate the level of local conservation. Exactly conserved sites are marked by asterisks.

Fig. 4 gives a short outline over the whole randomization procedure. We start by parametrization our model: we count the dinucleotides and calculate the corresponding stationary trinucleotide frequencies. A transition/transversion rate ratio for the alignment is estimated using maximum likelihood under a HKY+Γ model. Having set these parameters, we empirically estimate the relationship between substitutions and observed differences with equal rates for each site. This first estimate is used to calculate the site-specific rates, which are then used for the second estimation. In the next step, the pairwise distances between all sequences are calculated. For the calculation of the site-specific rates and the pairwise distances gap characters are treated in a special way as missing data (see Methods). From the distance matrix a tree is built using the BIONJ algorithm. An ancestral sequence is sampled from a first order Markov model parametrized according to the dinucleotide frequency in the original alignment. This is used as a starting sequence for the simulation that is guided by the tree. Finally, the gap pattern of the original alignment is introduced into the simulated one. Fig. 5 shows our rRNA example and two randomized versions obtained by this procedure.

### Implementation

We implemented our method in ANSI C in a program called SISSIz. The source code is available under the GNU Public Licence for download [48].

Some words on running time: One might suspect that the randomization algorithm including two times the sampling procedure to estimate the parameters of equation 9 and the maximum likelihood estimation of the transition/transversion rate ratio is relatively slow. Indeed, it is much slower than for example randomization by shuffling, but still very fast. To build the model for our example of 7 rRNAs of 158 length takes 0.2 seconds on a modern Intel Core 2 Quad CPU at 2.4 GHz. To simulate 1000 alignments using this model takes another 0.6 seconds.

### Testing

#### Randomizing vertebrate genomic alignments

We tested our randomization method on vertebrate genomic alignments. In a setting similar to recent genomic screens in vertebrates [11,20], we extracted Multiz [49] alignment blocks from human chromosome 1. We randomly selected 1000 alignment blocks between 70 and 120 nt in length and between 4 and 10 sequences without considering annotation information of any sort. These alignments are meant to represent an unbiased "genomic background" that may also contain functional elements like coding exons or structured RNAs depending on their frequency in the genome.

**Figure 6:**
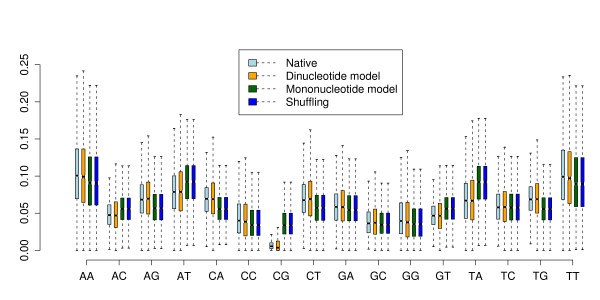
**Dinucleotide frequencies of genomic alignments.** 1000 vertebrate genome alignments were randomized using three different methods. The dinucleotide frequency of the native and randomized data is shown as box-plots.

The alignments were randomized using our new simulation procedure with both the dinucleotide and the mononucleotide model. In addition, we shuffled the alignments using shuffle-aln.pl. The global distribution of dinucleotides for the original and randomized data is shown in Fig. 6. As expected, the shuffling approach and the mononucleotide simulation give the same results. The dinucleotide distribution obtained by these methods, however, differs from the distribution in the native alignments. One can see for example the well known under-representation of CpGs in the native genomic data. Using our dinucleotide based model, we obtain simulated alignments which are statistically indistinguishable from the native data in terms of their average dinucleotide content.

**Figure 7:**
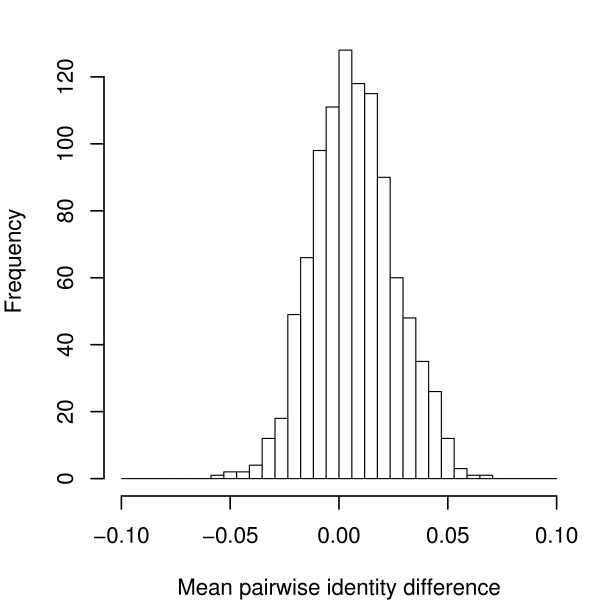
**Mean pairwise identity in randomized genomic alignments.** The distribution of the difference of the mean pairwise identity between the original genomic alignments and the simulated ones (dinucleotide model) is shown.

Also the observed sequence diversity of the simulated alignments closely follows the original data as shown in Fig. 7. 98.7% of the simulated alignments are within a range of ± 0.05 mean pairwise identity compared to the original alignments. It must be noted, that the distribution in Fig. 7 has a mean of +0.007 which indicates a subtle bias of the simulations towards higher MPIs. We suspect that this is an indirect result of the way we estimate site-specific rates and related to the issue of sites below saturation discussed before. However, this deviation does not have any practical consequences since it represents a conservative bias in the context of RNA folding controls and, more importantly, seems to be too small to have any noticeable effect at all.

#### Influence of randomization procedure on RNA predictions

**Figure 8:**
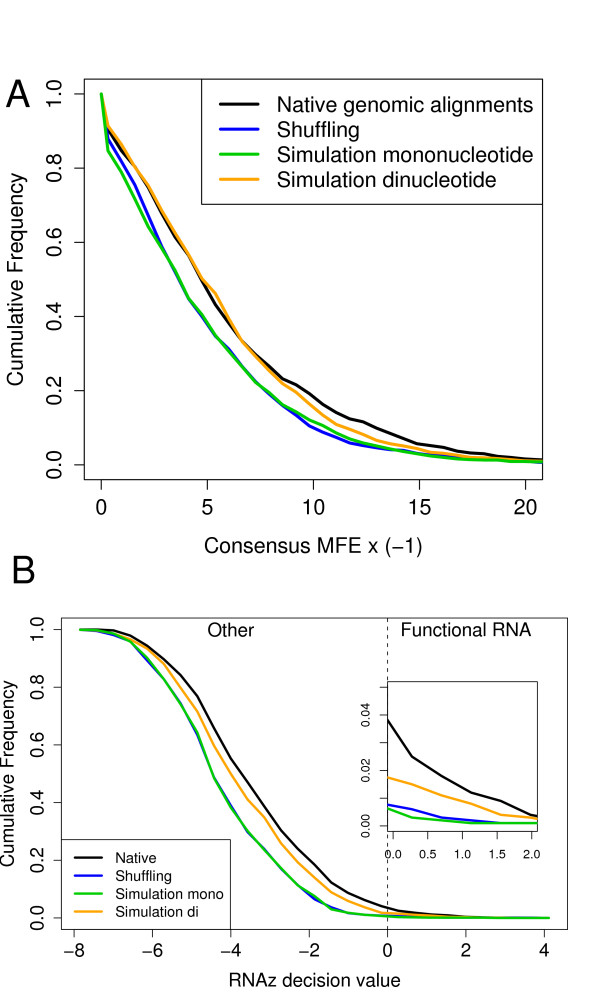
**Influence of the randomization procedure on RNA predictions.** (**A**) Cumulative frequency distribution of RNAalifold consensus folding energies for the native and randomized alignments. (**B**) Cumulative frequency distribution of RNAz scores. The "decision-value" is the result of the support vector machine classification. Positive values indicate a potential functional RNA while negative values indicate no significant fold. The positive tail is magnified.

The main motivation of this paper is to provide dinucleotide based controls for comparative RNA gene predictions. Therefore, we ran RNAalifold and RNAz on the alignments to demonstrate how different randomization procedures affect the results. Fig. 8A shows the distribution of RNAalifold consensus MFEs on the genomic alignments and their different randomizations. One can see that the genomic alignments show the most stable structures. There is a clear difference between the native genomic alignments and the shuffled and mononucleotide simulated ones. However, the folding energies of the dinucleotide simulated alignments are much closer to the native data. This difference between the di- and mononucleotide simulations reflects the bias caused by the genomic dinucleotide content. The difference between the native and the dinucleotide controls indicates the existence of RNA signals in the genome or, alternatively, another as yet unidentified bias.

Clearly, the differences shown here in these cumulative histograms might appear very subtle. The results for the RNAz predictions, however, show that such differences can strongly affect the statistics of RNA gene predictions (Fig. 8B). On this particular test set, RNAz predicts RNA signals in 4.3% of the native alignments. Using the conventional shuffling strategy or mononucleotide based simulation one would estimate a false positive rate of 0.8% or 0.7%, respectively. Using the more conservative dinucleotide based model the estimate would be 2.1%, i.e. three times higher. This is consistent with the results obtained by Babak *et al*. using their dinucleotide shuffling approach on pairwise alignments.

#### Calculating z-scores to predict structural RNAs

We can directly assess the significance of a predicted RNA by calculating a *z*-score. The folding energy of the native data *m *and the mean *μ *and standard deviation *σ *of randomized data is calculated. The significance of the native fold can then be expressed as *z *= (*m *- *μ*)/*σ*, i.e. the number of standard deviations from the mean. This score has been repeatedly used on single sequences applying mono- or dinucleotide shuffling or simulation using a zero or first order Markov model [23,24]. Using shuffled alignments as null model, this approach is implemented in the RNA gene finding program AlifoldZ [5]. The same strategy can be used in combination with our new dinucleotide base randomization strategy without any further modifications (Fig. 4).

To test the effectiveness of this approach, we conducted a benchmark similar to those used previously [5,6] for testing AlifoldZ and RNAz. We used multiple sequence alignments of eight different structural RNA families taken from the Rfam database [50]. The alignments contained three to six sequences and had a mean pairwise identity between 50% and 100% (see Methods for details). For the tests of AlifoldZ and RNAz, shuffled alignments were used as negative controls. For obvious reasons, this is not possible here. So we used genomic alignments from random locations of the human genome (see Methods). Using the "genomic background" as negative controls in this test implies the assumption that the genome does not contain any structural RNAs at all, which is clearly not valid. However, if we assume true structural RNAs to be sparse in the genome this conservative assumption seems to be a sensible choice.

We calculated *z*-scores with a sample size of 1000 randomizations for both sets of true structured RNAs and the genomic background using three different randomization methods: Shuffling (AlifoldZ), simulation using a mononucleotide model (SISSIz mono) and simulation using the dinucleotide model (SISSIz di). The results are summarized in Tab. 1.

**Table 1:** z-scores and classification performance

		RNAz			AlifoldZ			SISSIz (mono)			SISSIz (di)		
					
Data type	N	z	S_0.01_	S_0.05_	z	S_0.01_	S_0.05_	z	S_0.01_	S_0.05_	z	S_0.01_	S_0.05_
5S rRNA	368	n/a	0.77	0.98	-6.72	0.84	0.98	-6.35	0.86	0.98	-6.35	**0.93**	**1.00**
tRNA	382	n/a	0.74	0.98	-6.29	0.75	0.98	-6.24	0.74	0.98	-5.86	**0.88**	**0.99**
U2 snRNA	458	n/a	0.76	**1.00**	-7.17	0.89	0.99	-5.92	0.84	0.97	-5.22	**0.93**	0.99
U3 snRNA	377	n/a	0.52	**0.92**	-5.11	0.74	0.86	-4.47	0.69	0.83	-4.23	**0.76**	0.86
U5 snRNA	424	n/a	**0.90**	**0.96**	-5.61	0.77	**0.96**	-5.10	0.69	0.89	-4.43	0.76	0.91
Hammerhead	499	n/a	0.78	**1.00**	-6.68	0.85	**1.00**	-6.67	0.90	**1.00**	-6.66	**0.99**	**1.00**
Group II intron	480	n/a	0.68	**0.82**	-6.58	0.74	0.81	-6.77	0.72	0.81	-6.29	**0.77**	**0.82**
micro RNA precursor	571	n/a	0.75	**1.00**	-8.89	**1.00**	**1.00**	-8.84	**1.00**	**1.00**	-7.58	**1.00**	**1.00**

Total of all classes	3559	n/a	0.80	**0.96**	-6.75	0.87	0.95	-6.43	0.85	0.94	-5.93	**0.90**	0.95
Genomic background	3559	n/a	n/a	n/a	-0.44	n/a	n/a	-0.58	n/a	n/a	-0.15	n/a	n/a

S_0.01_, S_0.05 _... Sensitivity at a false positive rate of 0.01 and 0.05, respectively. The highest values among the four different methods are shown in bold letters.

Using mononucleotide based randomization the *z*-scores of the genomic background are approximately half a standard deviation from zero (-0.44 and -0.58, for shuffling and mononucleotide simulation respectively). This shows the relatively strong "bias" of the genomic background that causes false positive predictions as shown in the previous section and in reference 21. Albeit the signal does not vanish completely, the dinucleotide based *z*-scores are much closer to zero (-0.15).

The *z*-scores of the structural RNAs in this test set are on average well below -4 indicating a clear structural signal. Also here, we observe that mononucleotide simulated *z*-scores are lower than the dinucleotide simulated ones. In this case, a dinucleotide content that favors stable RNA structures is clearly not only a general background effect of the genomic base composition but a feature of structural RNAs. However, this signal is lost if the more conservative dinucleotide based null model is used.

There is also a clear difference between the two monunucleotide randomization procedures: Shuffling leads to more significant *z*-scores than simulation. The main reason is the fact that simulation results in higher standard deviations than shuffling which in turn lead to more conservative *z*-scores.

**Figure 9:**
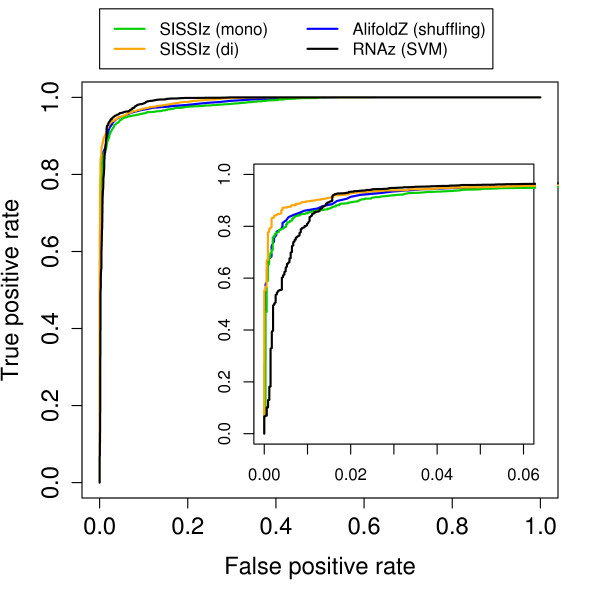
**Accuracy of *z*-score based classification of structured RNAs.** As positive examples, alignments from eight different classes of structural RNAs were used. As negative examples, random locations from genome wide vertebrate alignments were chosen. ROC curves are shown in dependence on the null model used. In addition, the results of the RNAz support vector machine are shown. The region of high specificity which is of special interest is magnified.

This shows that there are many effects that have to be taken into account. To assess the overall classification performance we generated receiver operating characteristic curves based on the three different *z*-scores, as well as the support vector machine score from RNAz (Fig. 9). In addition, we calculated the sensitivity at two different levels of specificity (0.01 and 0.05) for all four approaches (Tab. 1).

The ROC curve shows that all the methods perform very well on this test set. The curve further suggests that there is not much difference between them. However, differences become evident when looking at the region of high specificity, the only relevant region for practical applications (see inset Fig. 9). Here, the dinucleotide based approach generally outperforms the mononucleotide based methods. The improvement is small but clearly noticeable: At a false positive rate of 0.01%, dinucleotide based simulation shows the highest sensitivity for 7 of the 8 RNA classes. For example, in the tRNA group the sensitivity is 13% higher than AlifoldZ and RNAz. The latter performs significantly worse than all other methods at this level. At a false positive rate of 0.05%, dinucleotide simulation still performs slightly better than mononucleotide shuffling/simulation but is on the same level as RNAz that performs significantly better here.

## Discussion

Any experiment is only as good as its controls. What is true for experimental biology clearly also holds in the field of computational biology. The value of even the most sophisticated algorithm remains unclear if the significance of the results cannot be assessed properly. In this paper we addressed the problem of finding an adequate control strategy for comparative noncoding RNA predictions, which are started to get widely used for genome annotation.

Babak *et al*. demonstrated that currently used null models based on mononucleotide shuffling lead to an underestimation of the false positive rate in such screens. Although single opinions may be different [51], it is generally accepted that in the context of RNA gene prediction one should consider dinucleotide content as "background" rather than "signal". However, while there have been dinucleotide controlled randomization algorithms for single sequences for more than 20 years, it is a non-trivial problem in the case of multiple sequence alignments.

Here we devised a simulation procedure that produces alignments that have on average a given dinucleotide frequency and sequence diversity (globally and locally). The corresponding model needs to be relatively complex including overlapping dependencies and site-specific rates. Clearly, this model with a high number of parameters would not be a reasonable choice for use in phylogenetic analysis, but it turned out to be a good choice for this specific application.

We have to use heuristics and simplifications to estimate the tree and parameters for this model in reasonable time. The accuracy of our approach is measured in terms of how well the simulations reflect the properties of the original data. In this respect, we found that our strategy performs very well. Again, phylogenetic analysis was not the goal here, but some of the techniques introduced here might be of interest in this context. For example, we found that in the mononucleotide case our estimations for site-specific rates are surprisingly competitive when compared to the currently best maximum likelihood methods (data not shown).

The influence of the null model for genomic RNA predictions was found to be remarkable. Consistent with Babak and colleagues' findings on pairwise alignments, we observed three times more false positives using dinucleotide controls than using mononucleotide controls. This clearly shows that the new approach should be the method of choice to get more sensible estimates of the significance of comparative RNA predictions.

The next obvious step, is to use the new null model to improve current RNA gene prediction algorithms. In analogy to AlifoldZ, we combined our new simulation procedure with the RNAalifold consensus structure prediction algorithm. SISSIz calculates *z*-scores that are not biased by the genomic dinucleotide content and it is thus the first comparative gene finding program, that explicitely corrects for this effect. However, by using this conservative null model we also loose part of the signal in true structured RNAs. This might be the main reason, why the observed improvements in the overall classification performance were only relatively small.

In general, the support vector machine approach used by RNAz is preferable over the AlifoldZ approach, since it is orders of magnitude faster. However, it turned out to be difficult to create a dinucleotide based version of RNAz mainly for two reasons. Until now, there was no way to produce a dinucleotide controlled negative test set that is necessary for training the two class support vector machine [6]. With the method presented here, we have solved this problem and it is now possible to create test sets with specific dinucleotide properties. However, it remains an unsolved question how to compute dinucleotide based *z*-scores efficiently without shuffling. RNAz uses a regression approach to solve this problem for mononucleotides, which, unfortunately, does not scale well to the high dimensional dinucleotide case.

A promising alternative to the thermodynamic RNA prediction methods used in this paper, are probabilistic methods. The EvoFold algorithm [7] uses phylogenetic stochastic context-free grammars and, in its core, depends on a null model which is essentially an independent mononucleotide model. Since the folding grammar of EvoFold does not explicitely model stacking interactions there is no need for using a null model with overlapping dinucleotides as we have described here. However, also EvoFold was found to be affected to some degree by the dinucleotide content for reasons that are not immediately obvious [21]. A dinucleotide background model together with an advanced folding grammar that considers stacks can thus be expected to improve performance. However, it would require considerable effort to include such a null model into the sophisticated probabilistic framework of EvoFold.

Finally, we want to add that our randomization algorithm is not only of interest in the context of RNA gene prediction. It can be used for other comparative genomics applications whenever random alignments are needed as control. One could consider other applications in the context of RNA structures (e.g. prediction of conserved miRNA target sites) but also in different context (e.g. conserved sequence motifs). Currently our software implements a mono- and dinucleotide model which should be sufficient for many applications. In principle, however, it is also possible to consider higher order correlations within this framework.

## Methods

### Treating gaps

Gapped positions are treated as missing data. When counting the dinucleotide content, dinucleotides including a gap (N-, -N, --) are ignored. During simulation, gap positions are filled with nucleotides and gaps are re-introduced afterwards. Note that this way, if two nucleotides N_1 _and N_2 _are separated by a gap (e.g. N_1_----N_2_) the dinucleotide N_1_N_2 _is not in equilibrium. Depending on how gaps are treated in the downstream analysis this might be or might not be of concern. In any case, since not every gap position but only every gap *opened *is affected, this (potential) error is generally very small for reasonable alignments. So we did not consider correcting for this effect which would require reconstructing the gap history and setting lineage specific neighbourhood systems.

For calculating the site-specific rates, we also treat gaps as missing data and calculate ⟨*p*_*k*_⟩ in eq. 10 only over non-gap positions. After the simulation, the whole column has on average ⟨*p*_*k*_⟩ estimated from the non-gap positions that does not change when originally gapped positions are masked again. For calculating the observed differences *p *between two sequences we set positions that includes gaps to the average ⟨*p*_*k*_⟩ at this site.

### Distances above the level of saturation

When calculating genetic distances between two sequences the problem may occur that the observed number of differences is higher than the level of saturation. We found that this problem becomes severe when considering site-specific rates that generally lead to much lower levels of saturation (cf. Fig. 3B). We use a simple trick to overcome this limitation. We add additional sites during the simulation with site-specific rates that correspond to the average of the whole alignment (i.e. ⟨*p*_*k*_⟩ is set to 1-MPI in eq. 11 for all these additional sites). They act as "buffer sites" that reduces the number of mutation events that repeatedly hit the same sites of high rate leading to many double substitutions. As a consequence, the overall level of observed differences is higher and we do not run into problems building the distance matrix. In the end, the sites are removed again and since the relative rate ratios between the sites remained unchanged, we get the desired site-specific mutation patterns.

### Limiting base composition variation

During the testing of the influence of the randomization procedure on RNA folding, we made an interesting observation. As expected, the variance of the folding energies of randomized data is higher with simulation than with shuffling. However, we also observed that there is difference in the mean. Simulation leads to slightly higher (i.e. less stable) folding energies than shuffling. We observed this behaviour not only on multiple alignments but also on single sequences using shuffling vs. first order Markov simulation. We suspect that extreme deviations in the base composition that can occur in simulated data do not symmetrically lead to the same deviations of the folding energies but preferentially impair the formation of RNA structures. To compensate for this effect, we have introduced an option in our software that only outputs simulated alignments, that are within a specific range of mononucleotide frequencies. We can thus limit our simulations to mononucleotide frequencies that are almost exactly as in the original data. As a distance measure we use the Euclidean distance $\sum_{\alpha \in A,G,C,T}\sqrt{{\left(\pi_{\alpha }^{s}-\pi_{\alpha }\right)}^{2}}$ with *π*_*α *_the desired frequency of nucleotide *α *in the original alignment, and $\pi_{\alpha }^{s}$ the observed frequency in the simulation. For all the data shown in Figs. 8, 9 and Tab. 1 we used simulations with this cutoff set to 0.05.

### Software

For the simulations in Fig. 1 we used seq-gen version 1.3.2 [52,53]. Monunucleotide shuffling was carried out using shuffle-aln.pl with option "--mode conservative2". Together with alifoldz.pl it is available online [54]. For the tests in Figs. 1 and 8 we used RNAalifold from the Vienna RNA package [55] version 1.6.1. with options "-nc 0 -cv 0") and RNAz [56] version 1.0 with standard parameters. For implementation of our software we used a series of third party C-code that is available as open source: levmar [57] by Manolis Lourakis for least squares fitting, BIONJ [41,58] by Olivier Gascuel, PHYML [59,60] by Stéphane Guindon and Olivier Gascuel for maximum likelihood estimation of the transition/transversion rate, Vienna RNA package [55] by Ivo L. Hofacker and others for consensus folding in SISSIz.

### Sequence data

For the benchmark we used sequences from the following eight Rfam families: RF00001 (5S rRNA), RF00004 (U2 snRNA), RF00005 (tRNA), RF00008 (Hammerhead ribozyme), RF00012 (U3 snRNA), RF00020 (U5 snRNA), RF00029 (Group II intron), RF00104 (mir-10 precursor). From these sequences, a set of non-redundant alignments between 3 and 6 sequences per alignment and mean pairwise identity between appr. 50% and 100% was created as described [5,6]. The families were chosen because they represent different structural families and contain enough sequences to create sets of reasonable sample size.

Genomic alignments were extracted from Multiz 17-way vertebrate alignments available at the UCSC genome browser [61,62]. For creating the set of 1000 alignments used for Figs. 6 and 8, we used the rnazWindow.pl script from the RNAz software package [56,63] to get typical alignment blocks as used previously in genomic ncRNA screens [20] or [14]. For the benchmark we selected for each positive example of the structural RNA set a negative example from the genomic alignments. Subsets of sequences were chosen to get the same number of sequences and the same mean pairwise identity (± 0.05) as the structural RNA counterpart. Also the alignment length was adjusted accordingly (limited to a maximum length of 150).

## Authors' contributions

Both authors contributed equally.
